# Microscopic and computational analysis of the stomach, duodenum, and liver of rats with induced hyperprolactinemia treated with exogenous melatonin

**DOI:** 10.3389/fvets.2026.1709295

**Published:** 2026-05-14

**Authors:** João Vitor da Silva, Ryan Cristian da Silva, Renan Gabriel da Silva Ferreira, Alison José da Silva, Isaque Bertoldo Santos da Silva, Aline Maria Rodrigues dos Santos, Bruno José do Nascimento, Elba Verônica Matoso Maciel de Carvalho, Francisco Carlos Amanajás de Aguiar Júnior, Ewerton Fylipe de Araújo Silva, Priscilla Virgínio de Albuquerque, Apolônio Gomes Ribeiro, Bruno Mendes Tenorio, Lucas Rannier Ribeiro Antonino Carvalho, Fernanda das Chagas Angelo Mendes Tenorio

**Affiliations:** 1 Department of Histology and Embryology, Federal University of Pernambuco, Recife, Pernambuco, Brazil; 2 Department of Animal Morphology and Physiology, Federal University Rural of Pernambuco, Recife, Pernambuco, Brazil; 3 Department of Biochemistry, Federal University of Pernambuco, Recife, Pernambuco, Brazil; 4 Department of Histology, Federal University of Pernambuco, Vitória de Santo Antão, Pernambuco, Brazil; 5 Department of Anatomy, Federal University of Pernambuco, Vitória de Santo Antão, Pernambuco, Brazil; 6 Animal Science Department, Universidade Federal da Paraíba, Areia, Paraíba, Brazil; 7 Department of Physiology and Pharmacology, Karolinska Institutet, Stockholm, Sweden

**Keywords:** digestive system, fractal, histopathology, hyperprolactinemia, melatonin

## Abstract

**Introduction:**

This study evaluated the effects of exogenous melatonin (MEL) on morphological and computational alterations in the stomach, duodenum, and liver of hyperprolactinemic rats.

**Methods:**

Twenty-four animals were divided into three groups: Control (*n* = 8), Hyper (*n* = 8), and Hyper+MEL (*n* = 8). Hyperprolactinemia was induced with domperidone (4 mg/kg/day, subcutaneously [s.c.]), and MEL was administered at 200 μg/100 g (s.c.).

**Results:**

The Hyper group showed a higher body weight compared to the Control group, while both hyperprolactinemic groups exhibited gastric lumen dilation. PCNA immunolabeling increased in the liver and, to a lesser extent, in the stomach, while decreasing in the duodenum in the Hyper and Hyper+MEL groups. In the duodenum, both hyperprolactinemic groups exhibited increased villus and microvillus height, whereas a reduced Periodic Acid–Schiff (PAS)-stained area was detected only in the Hyper group. In the liver, microsteatosis, fewer Kupffer cells (KCs), and a higher number of binucleated hepatocytes were identified in hyperprolactinemic animals. Fractal analysis of goblet cells revealed no differences among groups. Serum MEL levels were elevated in the hyperprolactinemic groups, with the highest concentrations observed in the Hyper+MEL group.

**Discussion:**

In summary, hyperprolactinemia induced structural alterations in the stomach, duodenum, and liver, whereas MEL administration exerted limited effects, mainly influencing body weight and duodenal histochemical parameters, without consistent protective effects on the other evaluated outcomes.

## 1 Introduction

Prolactin (PRL) is primarily produced and secreted by lactotroph cells in the adenohypophysis (1) and is under strict neuroendocrine control, being mainly inhibited by hypothalamic dopamine. Elevated serum levels of this hormone result in a condition known as hyperprolactinemia (2).

Hyperprolactinemia is considered the most common endocrine alteration affecting the hypothalamic–pituitary axis. Its causes are diverse and can be classified as physiological, pharmacological, or pathological (2, 3). Physiological causes include pregnancy and breastfeeding (4); pharmacological causes are frequently associated with drugs such as antipsychotics, antidepressants, antihypertensives, and prokinetic agents; and pathological causes include systemic, hypothalamic, pituitary, and neurogenic disorders (2).

This condition occurs more frequently in women (5) and shows considerable prevalence in specific clinical contexts, ranging from 10 to 25% in women with amenorrhea or oligomenorrhea, approximately 30% in women with galactorrhea or infertility, and up to 75% in women presenting both galactorrhea and amenorrhea (6).

Beyond its reproductive role, PRL can influence the physiology of several organs, including those of the digestive system. Under physiological conditions, this hormone may contribute to hepatocyte renewal and growth processes (7) and promote intestinal mucosal expansion (8). However, elevated PRL levels may be associated with adverse effects, including the exacerbation of symptoms related to irritable bowel syndrome (9) and an increased risk of certain oncological pathologies (10). Despite these observations, the effects of sustained hyperprolactinemia on the morphology and cellular dynamics of digestive organs remain insufficiently understood.

Melatonin (MEL), a hormone mainly synthesized by the pineal gland and involved in neuroendocrine signaling and in the control of circadian rhythms, has also been described as an important modulator of gastrointestinal physiology. In addition to its pineal origin, MEL is produced in several gastrointestinal tracts and in the liver (11), indicating a broader role beyond central regulation. Due to its liposoluble nature, MEL readily crosses biological membranes, reaching different organs and intracellular compartments (12, 13). Consequently, several biological properties have been attributed to MEL, including antioxidant (14), anti-inflammatory (15), anti-aging (16), and anti-apoptotic effects (17).

MEL exerts its physiological actions through specific receptors, namely MT1, MT2, and MT3. In the gastrointestinal tract, receptors such as MT1 and MT3 have been identified in intestinal tissues, where they are associated with the regulation of gastrointestinal motility, inflammatory responses, and visceral pain (18). These receptor-mediated processes support the notion that MEL may play a protective or modulatory role in the digestive system under pathological conditions.

Although the systemic consequences of hyperprolactinemia have been widely described, its influence on the structure and cellular organization of the digestive system remains incompletely elucidated. At the same time, despite the recognized biological activities of MEL in the gastrointestinal tract, studies investigating its interaction with hyperprolactinemia are limited, and the potential effects of the exogenous application of this substance in this context have not yet been comprehensively characterized. Therefore, this study aimed to investigate how MEL administration affects microscopic and computational parameters in the stomach, duodenum, and liver of hyperprolactinemic rats.

## 2 Materials and methods

The present research project was approved by the Ethics Committee for Animal Use (CEUA) of the Federal University of Pernambuco (UFPE) under protocol number 23076/011943/2018–17.

### 2.1 Experimental animals

Twenty-four albino rats of the Wistar lineage (*Rattus norvegicus* albinus), aged 90 days, were used, sourced from the Animal Facility of the Academic Center of Vitória (CAV), UFPE. The animals were kept in the experimental Animal Facility of CAV in polypropylene cages, stored in rooms under standardized laboratory conditions: luminosity of 60 lux and a 12-h light–dark cycle; temperature (22 °C ± 1 °C); relative air humidity (45% ± 5%); and an exhaust system with air renewal. All animals were provided with water and standard feed from the animal facility (Labina, Presence^®^, *ad libitum*).

Only male rats were used to reduce the variability associated with the estrous cycle of females, which could interfere with the parameters evaluated.

Adult male rats were randomly divided into three groups, each consisting of eight animals, namely: Control group—without treatment; Hyper group—rats induced with hyperprolactinemia (domperidone); Hyper+MEL group—rats induced with hyperprolactinemia (domperidone) and treated with MEL.

### 2.2 Induction of hyperprolactinemia

Induction of hyperprolactinemia was performed via subcutaneous injection of domperidone (DOMP) (Sigma, St. Louis, MO, USA) at a daily dose of 4 mg/kg, always at 11 a.m., for 60 days. DOMP was dissolved in 1 mL of saline solution (NaCl 0.9%) (19).

### 2.3 Treatment with melatonin

The treatment with MEL (Sigma, St. Louis, MO, USA) was carried out via subcutaneous injection, to slow down absorption by the body, at a daily dose of 200 μg per 100 g of body weight of the animal, always at 6 p.m., for 60 days. MEL was dissolved in a volume of ethanol (0.02 mL) and diluted in saline solution (NaCl 0.9%). This dosage and treatment duration were chosen based on previously validated protocols that demonstrated the effectiveness of this regimen in modulating endocrine parameters in rats (19).

### 2.4 Analysis of animal weight

The animals from all groups were weighed daily during the 60-day experiment. An analytical precision balance, Celtac FA2104N, was used for weight measurement.

### 2.5 Histopathological and histochemical analysis

After 60 days of experimentation, the animals were subjected to terminal anesthesia via intraperitoneal injection of urethane (10%) and chloralose (0.4%), totaling 10 mL/kg (1 g/kg urethane + 40 mg/kg chloralose), and euthanized by lethal effect, in accordance with previous studies (20, 21). The stomach, duodenum, and liver were then collected and immediately immersed in 10% buffered formalin for 48 h. Subsequently, the samples were processed using the standard paraffin embedding technique. Transverse sections of the stomach, duodenum, and liver were obtained, dehydrated in increasing concentrations of ethyl alcohol (70, 80, 90%, and absolute), clarified in xylene, impregnated, and embedded in paraffin. For each animal, eight slides per organ were prepared from 3 μm histological sections cut with a microtome (Leica, Germany). Stomach and liver slides were stained with Hematoxylin and Eosin (H&E), whereas duodenal slides were stained with both H&E and Periodic Acid–Schiff (PAS). Histological sections were taken from comparable anatomical regions of each organ to ensure methodological consistency. All preparations were analyzed under a light microscope (NIS Elements Nikon^®^) using the National Toxicology Program as a parameter and photomicrographed with a photomicroscope (Nikon Eclipse 80i).

In the histochemical analysis, photomicrographs of the duodenum were used to calculate the PAS labeling area using ImageJ software (version 1.44; Research Services Branch, U.S. National Institutes of Health, Bethesda, MD, USA). The images were segmented according to the macro: rename(“a”); run(“Colour Deconvolution,” “vectors = [H PAS]”); selectWindow(“a-(Colour_3)”); selectWindow(“a- (Colour_2)”); close(); selectWindow(“a-(Colour_1)”); close(); selectWindow(“Colour Deconvolution”); close(); run(“8-bit”); run(“Auto Threshold,” “method = Minimum ignore_black white”); run(“Median.,” “radius = 6”); run(“Analyze Particles.,” “size = 20-Infinity show = [Masks] display clear”). Thus, the PAS-positive labeling area was calculated from 20 microscopic fields per slide for each animal at 400 × magnification. Histopathological and histochemical evaluations were carried out by a researcher blinded to the experimental groups to avoid assessment bias.

### 2.6 Histomorphometric analysis

For the morphometric analysis of the stomach, duodenum, and liver, microscopic fields were selected using systematic random sampling to provide adequate coverage of each histological section while avoiding overlapping areas. A predefined number of fields was analyzed for each animal across all experimental groups. In the stomach, 10 fields per slide were imaged at 100 × magnification to measure gastric pit height, whereas 20 fields at 400 × magnification were used to assess epithelial height and gland diameter. In the duodenum, villus height and muscular layer thickness were measured in 10 fields at 40 × magnification, while 20 fields at 400 × magnification were examined to determine crypt diameter, epithelial height, apical brush border (microvilli) height, and goblet cell number. In the liver, Kupffer cells (KCs) and binucleated hepatocytes were quantified in 20 fields at 400 × magnification. Image analysis was performed using ImageJ software (version 1.44; Research Services Branch, U.S. National Institutes of Health, Bethesda, MD, USA). All morphometric analyses were conducted by an investigator masked to group assignments to ensure unbiased results.

### 2.7 Fractal dimension (FD) and the lacunarity method

The FD of duodenal goblet cells and hepatic sinusoids was calculated using the box-counting method (Dbox) in ImageJ software (version 1.44; Research Services Branch, U.S. National Institutes of Health, Bethesda, MD, USA). In this procedure, photomicrographs were overlaid with boxes of varying sizes (r), and the number of boxes N(r) containing at least one point of the analyzed structure was recorded. The log–log plot of N(r) as a function of r yielded the slope corresponding to the FD. Additionally, lacunarity was calculated to assess the heterogeneity of empty spaces, providing a complementary measure to FD. For this analysis, grids with different box sizes (*ε*, representing box size) and orientations (g, representing grid orientation) were applied.

### 2.8 Immunohistochemical analysis (cell nuclear proliferation antigen—PCNA)

For cell proliferation analysis, the silanized slides containing histological sections of the stomach, duodenum, and liver were deparaffinized and rehydrated in xylene and alcohol, respectively. Antigen retrieval was performed using a citrate buffer solution (pH 6.0) heated in a microwave for 5 min. Endogenous peroxidase was inhibited using a solution of hydrogen peroxide (3%) in distilled water for 30 min. Non-specific binding was blocked by incubating the slides in phosphate-buffered saline (PBS) and bovine serum albumin (BSA) (5%) for 1 h. The anti-PCNA antibody (Sigma-Aldrich, Brazil) was diluted in PBS/BSA (1%) and incubated for 1 h. Subsequently, the slides were treated with a Histofine® secondary antibody for 30 min. The antigen–antibody reaction was observed under a light microscope, resulting in a brown staining precipitate after the application of 3,3′-diaminobenzidine (DAB) for 6 min.

In the immunohistochemical analysis, photomicrographs of the stomach, duodenum, and liver were used to calculate the cell proliferation area using ImageJ software (version 1.44; Research Services Branch, U.S. National Institutes of Health, Bethesda, MD, USA). The images were segmented following the protocol below: image selection, image, color, color deconvolution, FastRed-FastBlue-DAB, close red and blue components, process, contrast enhancement (0.6% saturation), image, adjust, threshold Otsu red apply, process, median filter (5–6 pixels), analyze, and histogram list. The median filter was used to reduce small pixel artifacts resulting from staining that could interfere with the analysis. Thus, the cell proliferation area was calculated from a total of 20 fields on each slide per animal under 400 × magnification. Immunohistochemical evaluation was performed by a researcher blinded to the experimental groups to ensure impartial interpretation.

### 2.9 Hormonal assay

For the hormonal assay of PRL and MEL, 1 mL of blood was obtained via cardiac puncture (22) between 8:00 a.m. and 10:00 a.m. to minimize circadian variation. The collected blood was placed in dry and heparinized tubes for serum and plasma separation, respectively. Subsequently, the samples were centrifuged under refrigeration, and the supernatant was transferred to Eppendorf tubes in duplicate and stored at −80 °C until hormone analysis. Serum PRL levels were measured using a Rat Prolactin ELISA Kit (Invitrogen, Thermo Fisher Scientific, San Diego, CA, USA; Cat. No. ERA50RB), with a sensitivity of ~0.41 ng/mL and a detection range of ~0.41–100 ng/mL. Serum MEL levels were measured using a Rat Melatonin ELISA Kit (Cusabio Biotech, Wuhan, China; Cat. No. CSB E13433r), with a sensitivity of 12.5 pg./mL and a detection range of 12.5–500 pg./mL. All samples were analyzed in duplicate, and the mean of the two readings was considered the final value for each sample. Intra-assay variability was monitored, with coefficients of variation consistently <10%.

### 2.10 Statistical analyses

Statistical analyses were conducted using GraphPad Prism version 8.0 (GraphPad Software, San Diego, CA, USA). Data were presented as median and interquartile range (IQR). To evaluate variation among groups, the non-parametric Kruskal–Wallis test was applied, followed by Dunn’s *post-hoc* test for multiple comparisons. Statistical significance was defined at *p* < 0.05. Correlation analyses between hormonal and cellular variables were not included, since the primary objective of the study was to compare morphological and proliferative outcomes across groups.

## 3 Results

### 3.1 The analysis of animal weight

The hyper group exhibited a significant increase in body weight in comparison to the Control group, an effect that was substantially reversed following MEL treatment in the Hyper+MEL group (Table 1).

**Table 1:** Body weight during the treatment period in experimental groups (*n* = 8 per group).

Parameter/Groups	Control	Hyper	Hyper+MEL	*p*
Body weight	340 (315–365)a	360 (345–375)b	338 (328–347)a	0.00*

Values are presented as median (IQR, g).

*Different letters denote significant differences between groups (Kruskal–Wallis test followed by Dunn’s *post-hoc* test, *p* ≤ 0.05). Groups: Control—untreated, Hyper—hyperprolactinemia model, Hyper+MEL—hyperprolactinemia treated with melatonin.

### 3.2 Histopathological analysis

#### 3.2.1 Stomach

**Figure 1:**
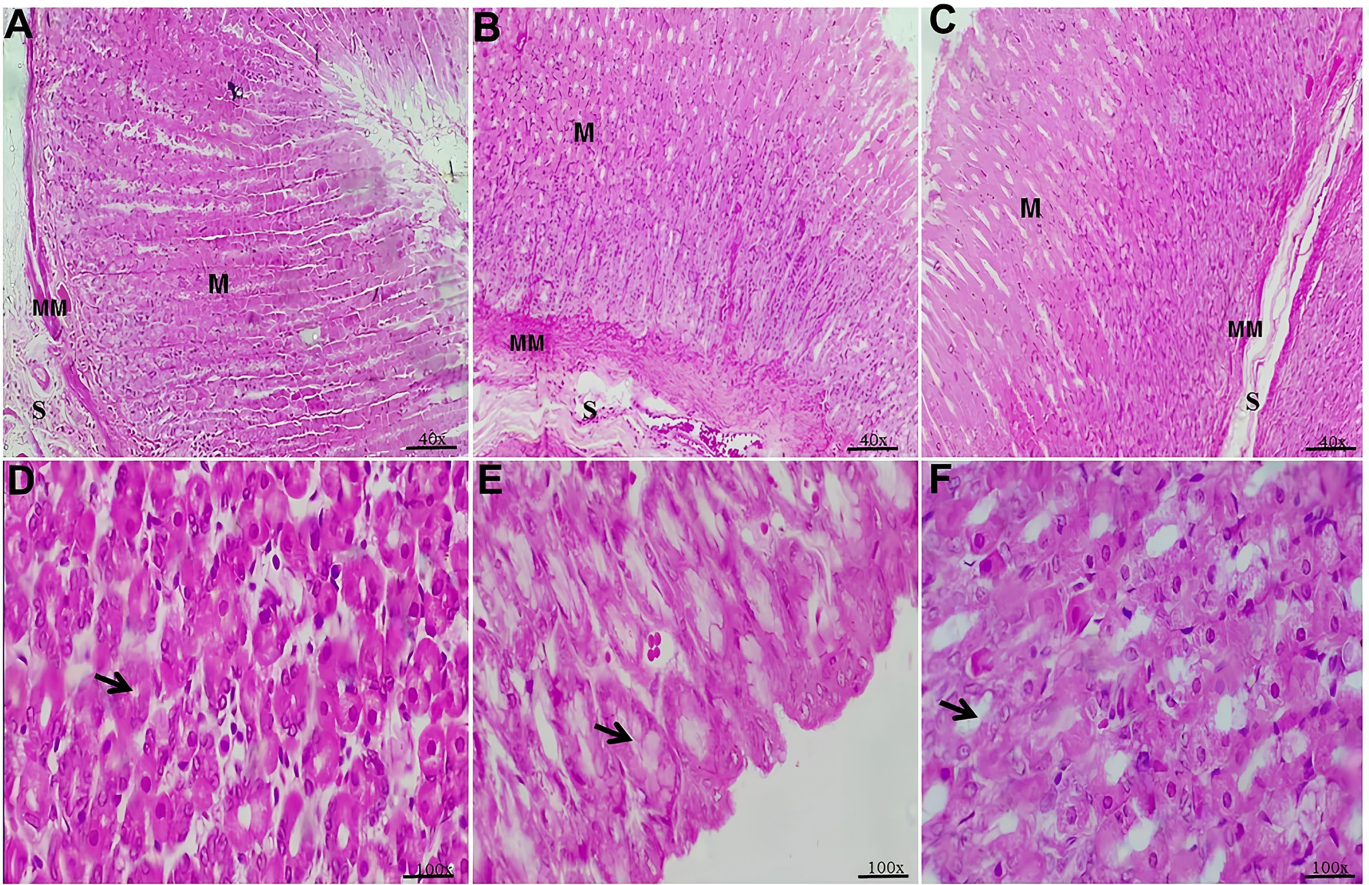
Photomicrographs of the stomach from the experimental groups. **(A)** Control group showing the mucosal layer (M), muscularis mucosae (MM), and submucosa (S). **(B)** Hyper group showing the mucosal layer (M), muscularis mucosae (MM), and submucosa (S). **(C)** Hyper+MEL group showing the mucosal layer (M), muscularis mucosae (MM), and submucosa (S) (40×). **(D)** Control group demonstrating the glandular lumen (arrow). **(E)** Hyper group with dilation of the glandular lumen (arrow). **(F)** Hyper+MEL group with dilation of the glandular lumen (arrow) (100×). Sections were stained with HE. Scale bars = 50 μm (40×) and 20 μm (100×).

The histological analysis of the stomach across all studied groups revealed well-defined mucosal, muscularis mucosae, and submucosal layers. However, in the mucosal layer of the Hyper and Hyper+MEL groups, a dilation of the glandular lumen was observed compared to the Control group (Figure 1).

#### 3.2.2 Duodenum

**Figure 2:**
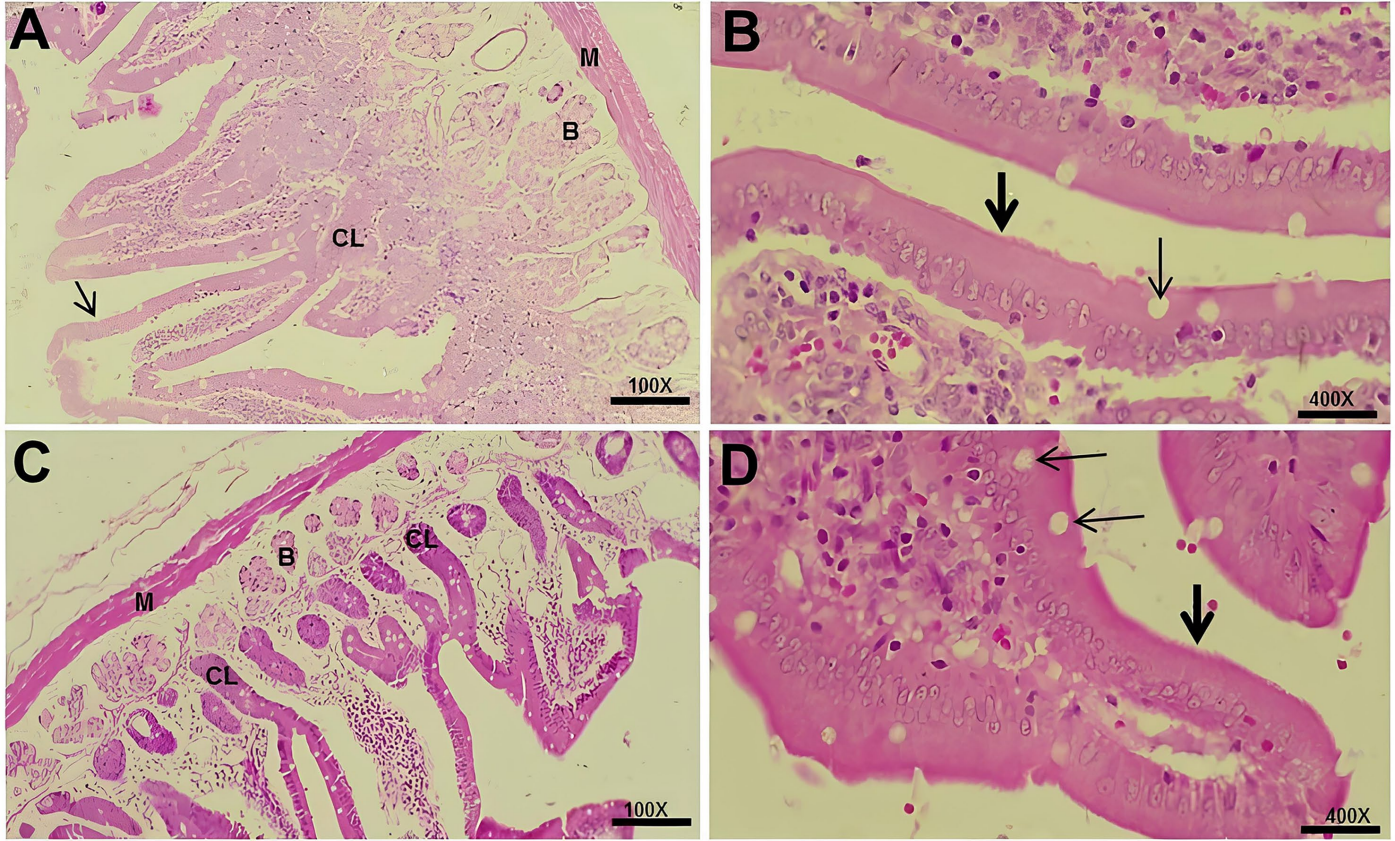
Photomicrographs of the duodenum from the experimental groups. **(A)** Control group showing simple columnar epithelium with absorptive cells (arrow), Lieberkühn crypts (CL), Brunner’s glands (B), and muscular layer (M) (100×). **(B)** Control group showing goblet cells (thin arrow) and microvilli (thick arrow) (400×). **(C)** Hyper group showing Lieberkühn crypts (CL), Brunner’s glands (B), and muscular layer (M) (100×). **(D)** Hyper+MEL group showing goblet cells (thin arrow) and epithelium with microvilli (thick arrow) (400×). Sections were stained with HE. Scale bars = 20 μm (100×) and 10 μm (400×).

Histological examination of the duodenum demonstrated preserved mucosal, submucosal, and muscular layers in all experimental groups, with intestinal villi lined by simple columnar epithelium containing absorptive and goblet cells. No relevant histopathological alterations were observed among the groups studied (Figure 2).

#### 3.2.3 Liver

**Figure 3:**
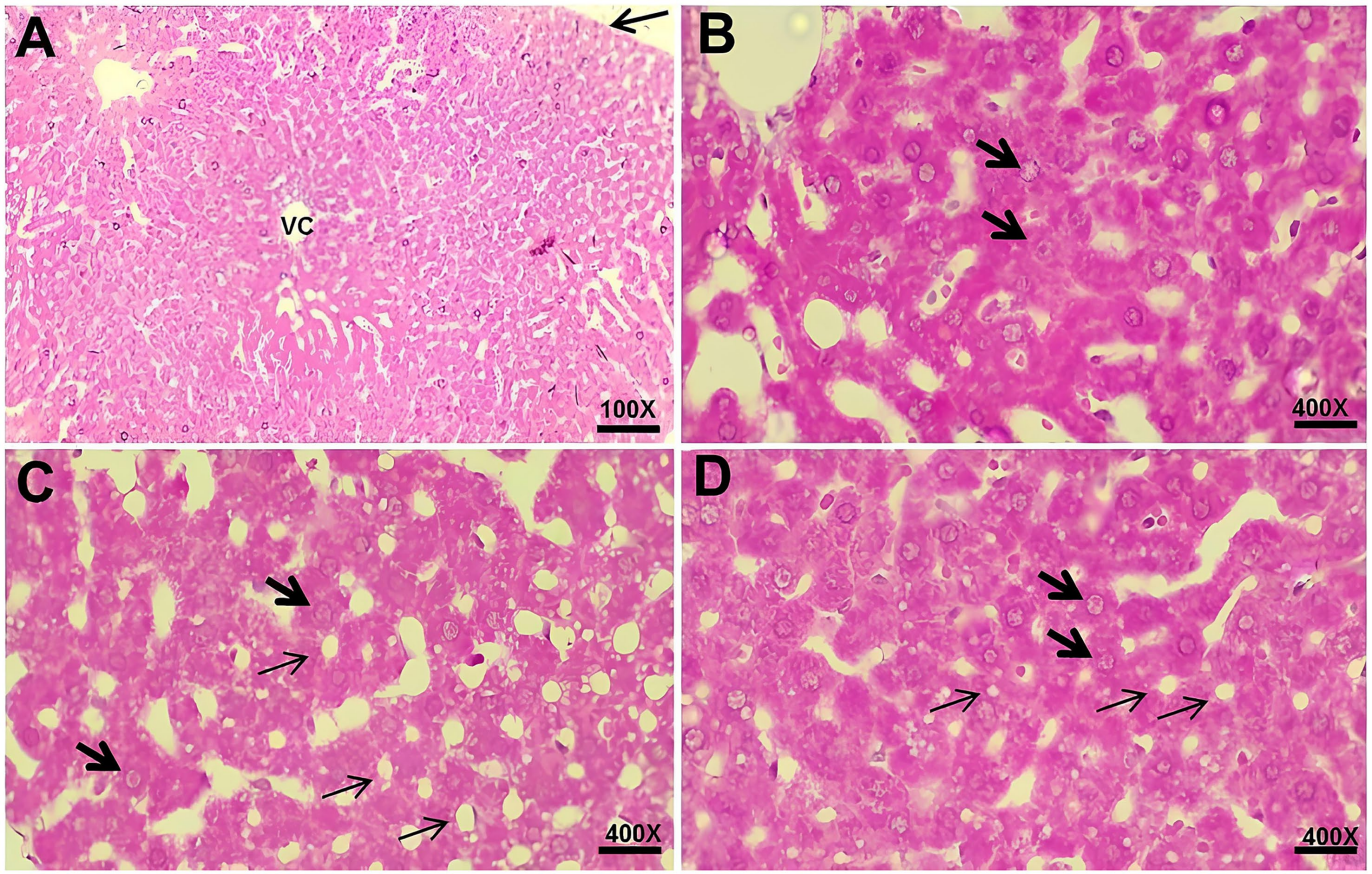
Photomicrographs of the liver from the experimental groups. **(A)** Control group showing connective tissue capsule (arrow) and central lobular vein (CLV) (100×). **(B)** Control group with hepatocytes (arrow). **(C)** Hyper group showing hepatocytes (thick arrow) and microsteatosis (thin arrows). **(D)** Hyper+MEL group showing hepatocytes (thick arrow) and microsteatosis (thin arrows) (400×). Sections were stained with HE. Scale bars = 20 μm (100×) and 10 μm (400×).

The histological analysis of the liver in all studied groups showed a thin capsule of connective tissue externally and lobules containing veins of various calibers. Additionally, the parenchyma exhibited hepatocytes arranged radially around the central lobular vein. However, the Hyper and Hyper+MEL groups exhibited microsteatosis when compared to the Control group (Figure 3).

### 3.3 Histochemical analysis

**Figure 4:**
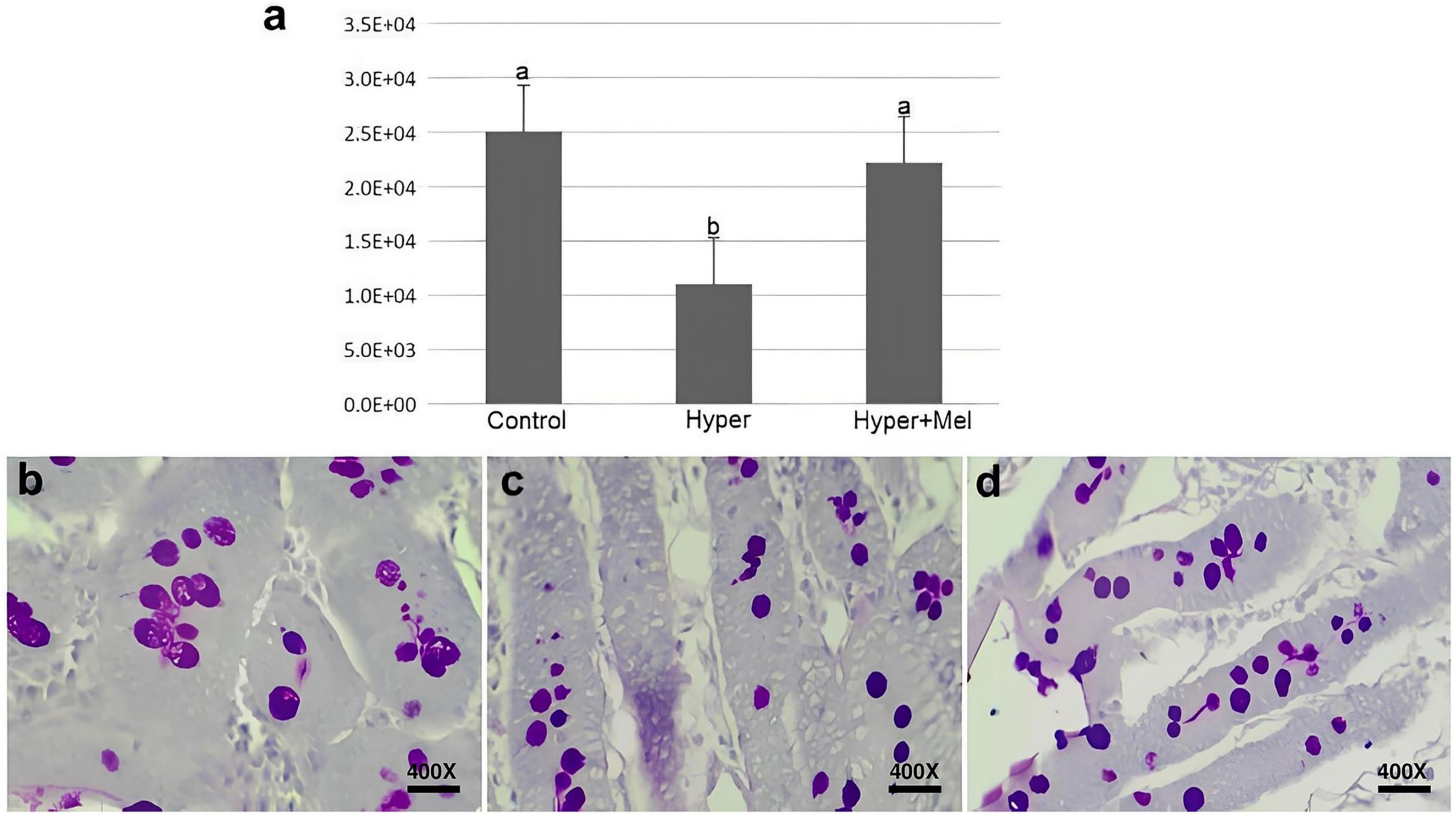
PAS staining of duodenum sections in experimental groups (*n* = 8 per group). **(a)** Quantitative analysis of PAS-positive area (*p* = 0.001). **(b–d)** Representative photomicrographs: **(b)** Control, **(c)** Hyper, and **(d)** Hyper+MEL. *Different letters indicate statistically significant differences (Kruskal–Wallis test followed by Dunn’s *post-hoc* test, *p* ≤ 0.05). Images acquired at 400 × magnification. Scale bar = 10 μm.

The analysis of PAS staining areas in the duodenum revealed a significant reduction in the Hyper group compared to the Control group. Nevertheless, in the presence of exogenous MEL, this decrease was considerably reversed (Figure 4).

### 3.4 Histomorphometric analysis

#### 3.4.1 Stomach

The histomorphometric analysis of the stomach demonstrated that the height of the epithelium and gastric pits did not differ significantly among the studied experimental groups. However, the glandular diameter was greater in the Hyper and Hyper+MEL groups compared with the Control group (Table 2).

**Table 2:** Stomach morphometry in experimental groups (*n* = 8 per group).

Parameters/Groups	Control	Hyper	Hyper+MEL	*p*
Epithelium height	7.4 (6.8–8.1)	7.4 (6.9–8.2)	7.5 (6.9–8.1)	0.88
Pit height	65.0 (58–73)	64.1 (58–70)	64.6 (59–71)	0.70
Gland diameter	15.0 (11–20)a	16.3 (13–19)b	16.1 (13–19)b	0.001*

Values are presented as median (IQR, μm).

*Different letters denote significant differences between groups (Kruskal–Wallis test followed by Dunn’s *post-hoc* test, *p* ≤ 0.05). Groups: Control—untreated, Hyper—hyperprolactinemia model, Hyper+MEL—hyperprolactinemia treated with melatonin.

#### 3.4.2 Duodenum

Histomorphometric assessment of the duodenum revealed increased villus and microvillus height in the Hyper and Hyper+MEL groups compared to the Control group, whereas epithelial height, muscular layer diameter, crypt diameter, and goblet cell count showed no significant differences (Table 3).

**Table 3:** Duodenum morphometry in experimental groups (*n* = 8 per group).

Parameters/Groups	Control	Hyper	Hyper+MEL	*p*
Villus height	129 (92–160)a	142 (110–170)b	178 (135–220)b	0.01*
Epithelium height	25.3 (22–28)	27.3 (24–31)	24.3 (21–28)	0.06
Microvillus height	1.2 (0.8–1.6)a	1.8 (1.4–2.2)b	2.0 (1.6–2.4)b	0.02*
Diameter of the muscular layer	22.8 (20–25)	21.7 (16–27)	24.4 (21–28)	0.10
Diameter of the crypts	33.0 (29–37)	32.8 (28–38)	34.6 (30–39)	0.49
Count of goblet cells	23 (14–32)	20 (12–28)	24 (15–32)	0.58

Values are presented as median (IQR). Morphometric parameters are expressed in μm, and goblet cell counts are presented as the number of cells.

*Different letters denote significant differences between groups (Kruskal–Wallis test followed by Dunn’s post-hoc test, *p* ≤ 0.05). Groups: Control—untreated, Hyper—hyperprolactinemia model, Hyper+MEL—hyperprolactinemia treated with melatonin.

#### 3.4.3 Liver

The histomorphometric analysis of the liver exhibited a lower quantity of KCs in the Hyper and Hyper+MEL groups compared to the Control group. Additionally, it was verified that there was a higher number of binucleated hepatocytes in the Hyper+MEL group compared to the Control and Hyper groups (Table 4).

**Table 4:** Liver morphometry in experimental groups (*n* = 8 per group).

Parameters/Groups	Control	Hyper	Hyper+MEL	*p*
Kupffer cells	15 (10–21)a	14 (10–18)b	12 (8–16)b	0.001*
Binucleated hepatocytes	5.2 (3–7)a	6.6 (4–9)a	9.1 (7–11)b	0.001*

Values are presented as median (IQR). Cellular counts (Kupffer cells and binucleated hepatocytes) are presented as the number of cells.

*Different letters denote significant differences between groups (Kruskal–Wallis test followed by Dunn’s *post-hoc* test, *p* ≤ 0.05). Groups: Control—untreated, Hyper—hyperprolactinemia model, Hyper+MEL—hyperprolactinemia treated with melatonin.

### 3.5 Analysis of FD and lacunarity

#### 3.5.1 Duodenum

**Figure 5:**
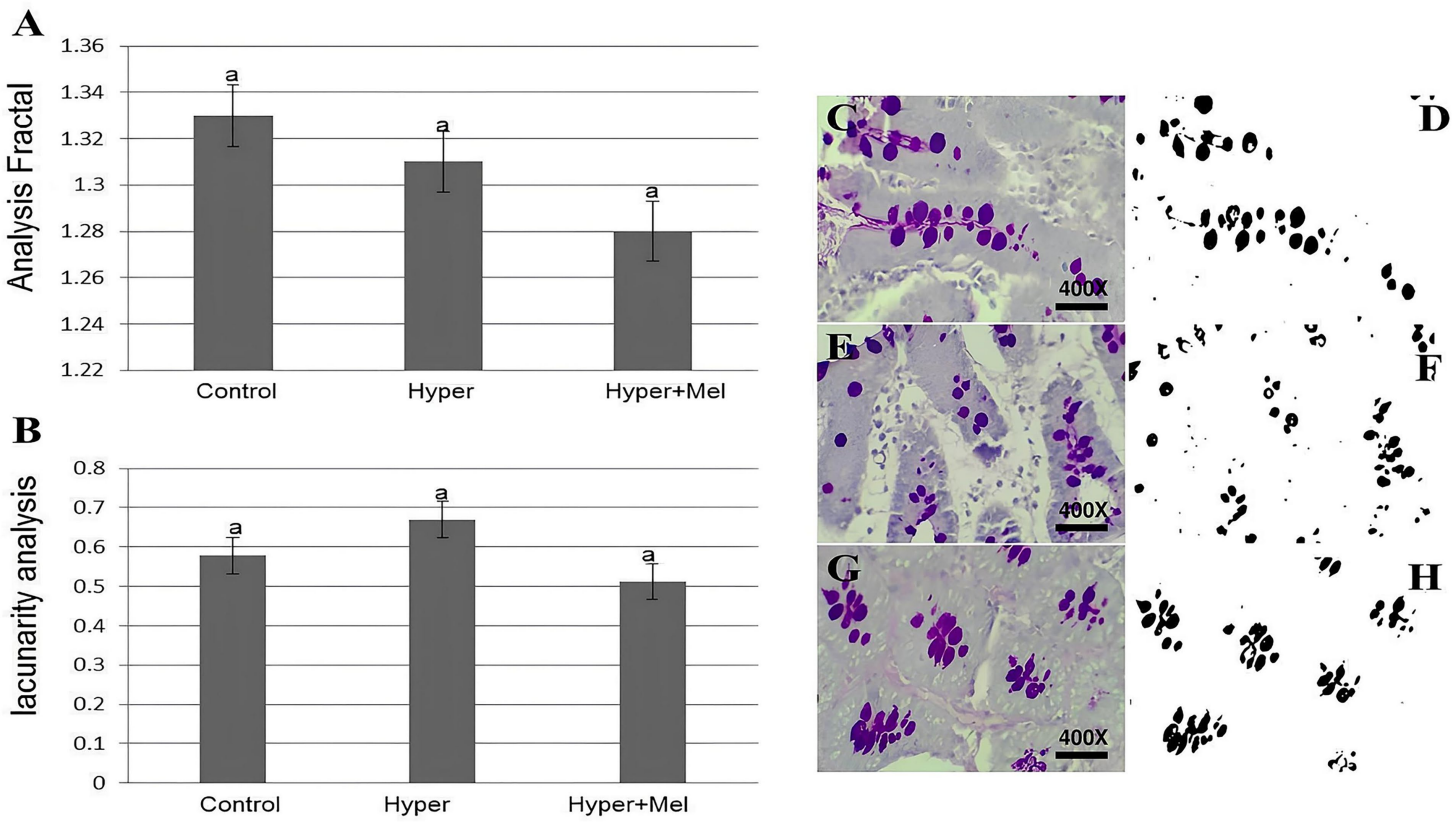
FD and lacunarity of duodenal goblet cells in experimental groups (*n* = 8 per group). **(A)** FD of duodenal goblet cells (*p* = 0.31). **(B)** Lacunarity of duodenal goblet cells (*p* = 0.44). **(C–H)** Photomicrographs and segmentation of the duodenum from the experimental groups. **(C,D)** Control group. **(E,F)** Hyper group. **(G,H)** Hyper+MEL group. Photomicrographs **(C,E,G)** stained with PAS. Images **(D,F,H)** processed using ImageJ software. *Different letters indicate statistically significant differences (Kruskal–Wallis test followed by Dunn’s *post-hoc* test, *p* ≤ 0.05). Images acquired at 400 × magnification. Scale bar = 10 μm.

Fractal evaluation and lacunarity of duodenal goblet cells revealed no statistically significant differences among the experimental groups studied (Figure 5).

#### 3.5.2 Liver

**Figure 6:**
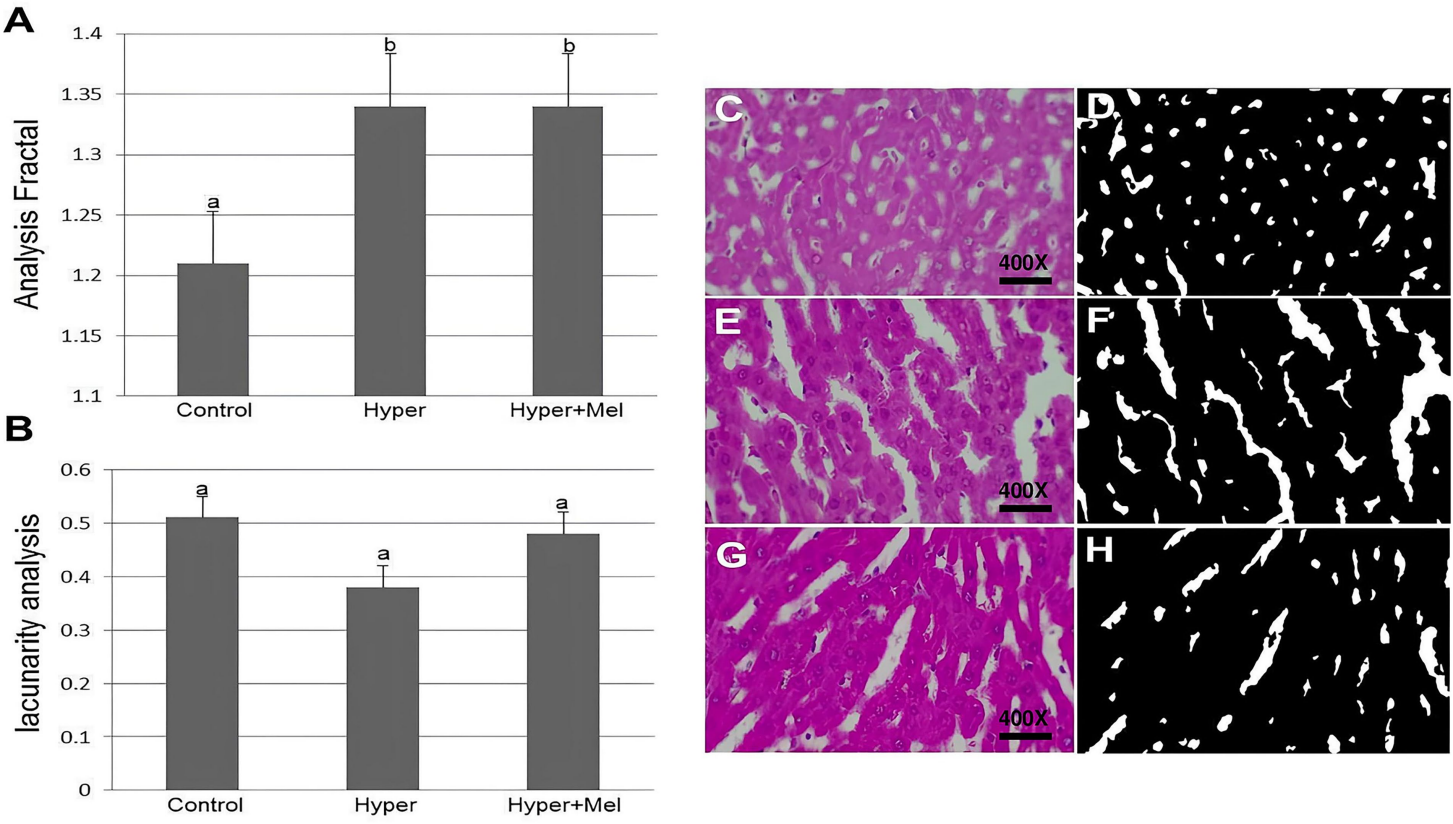
FD and lacunarity of hepatic sinusoids in experimental groups (*n* = 8 per group). **(A)** FD of hepatic sinusoids (*p* = 0.04). **(B)** Lacunarity of hepatic sinusoids (*p* = 0.07). **(C–H)** Photomicrographs and segmentation of the liver from the experimental groups. **(C,D)** Control group. **(E,F)** Hyper group. **(G,H)** Hyper+MEL group. Photomicrographs **(C,E,G)** stained with HE. Images **(D,F,H)** processed using ImageJ software. *Different letters indicate statistically significant differences (Kruskal–Wallis test followed by Dunn’s *post-hoc* test, *p* ≤ 0.05). Images acquired at 400 × magnification. Scale bar = 10 μm.

The FD of hepatic sinusoids was significantly higher in the Hyper and Hyper+MEL groups than the Control group, whereas lacunarity demonstrated no significant differences among the groups (Figure 6).

### 3.6 Cell proliferation analysis

#### 3.6.1 Stomach

**Figure 7:**
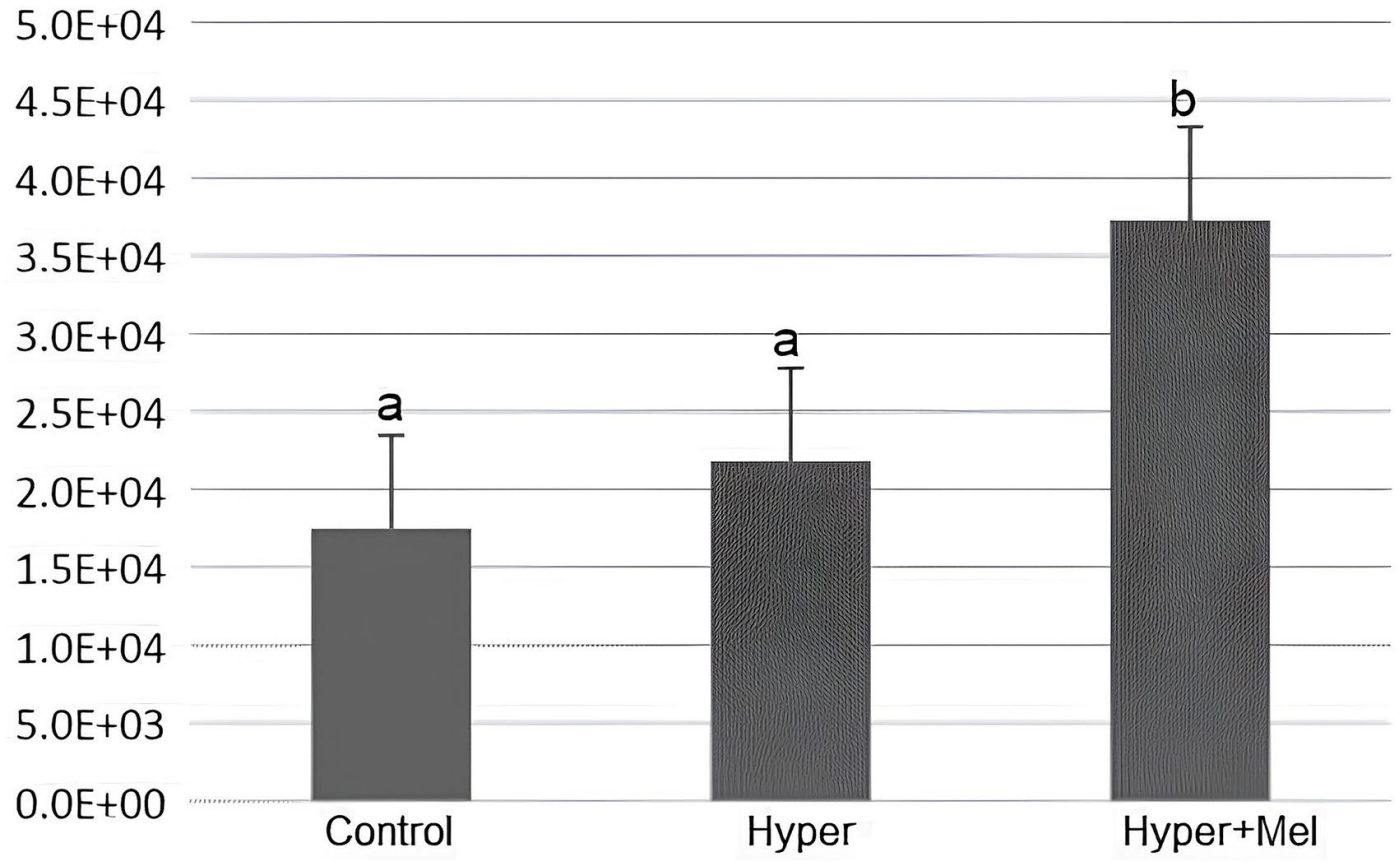
Analysis of PCNA in the stomach of the experimental groups (*p* = 0.011). *Different letters indicate statistically significant differences (Kruskal–Wallis test followed by Dunn’s *post-hoc* test, *p* ≤ 0.05).

Analysis of PCNA in the stomach showed a significant increase in the Hyper+MEL group compared to the Control and Hyper groups (Figure 7).

#### 3.6.2 Duodenum

**Figure 8:**
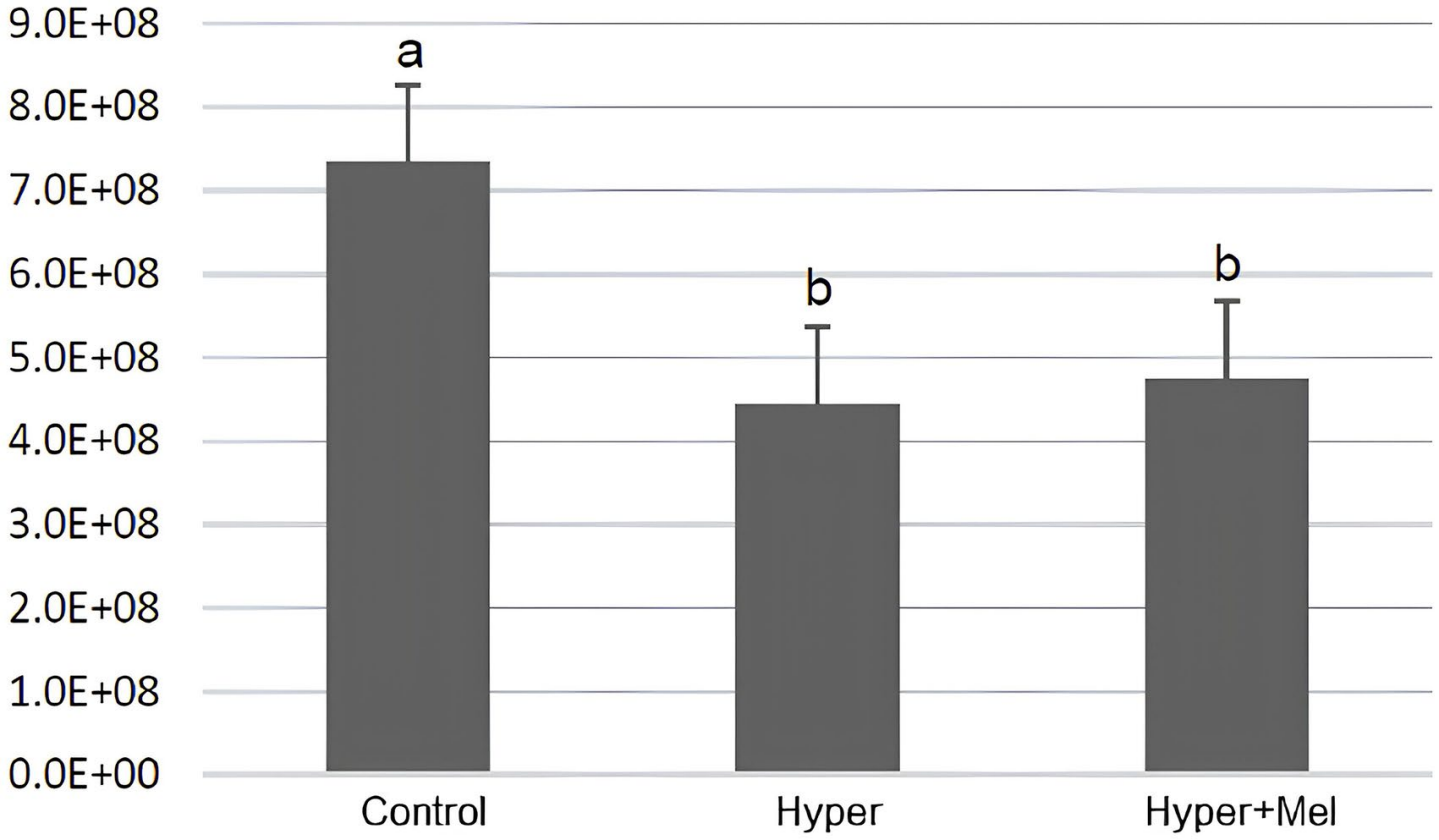
Analysis of PCNA in the duodenum of the experimental groups (*p* = 0.002). *Different letters indicate statistically significant differences (Kruskal–Wallis test followed by Dunn’s *post-hoc* test, *p* ≤ 0.05).

Immunohistochemical analysis of the duodenum indicated that the Hyper and Hyper+MEL groups showed reduced PCNA immunolabeling compared to the Control group (Figure 8).

#### 3.6.3 Liver

**Figure 9:**
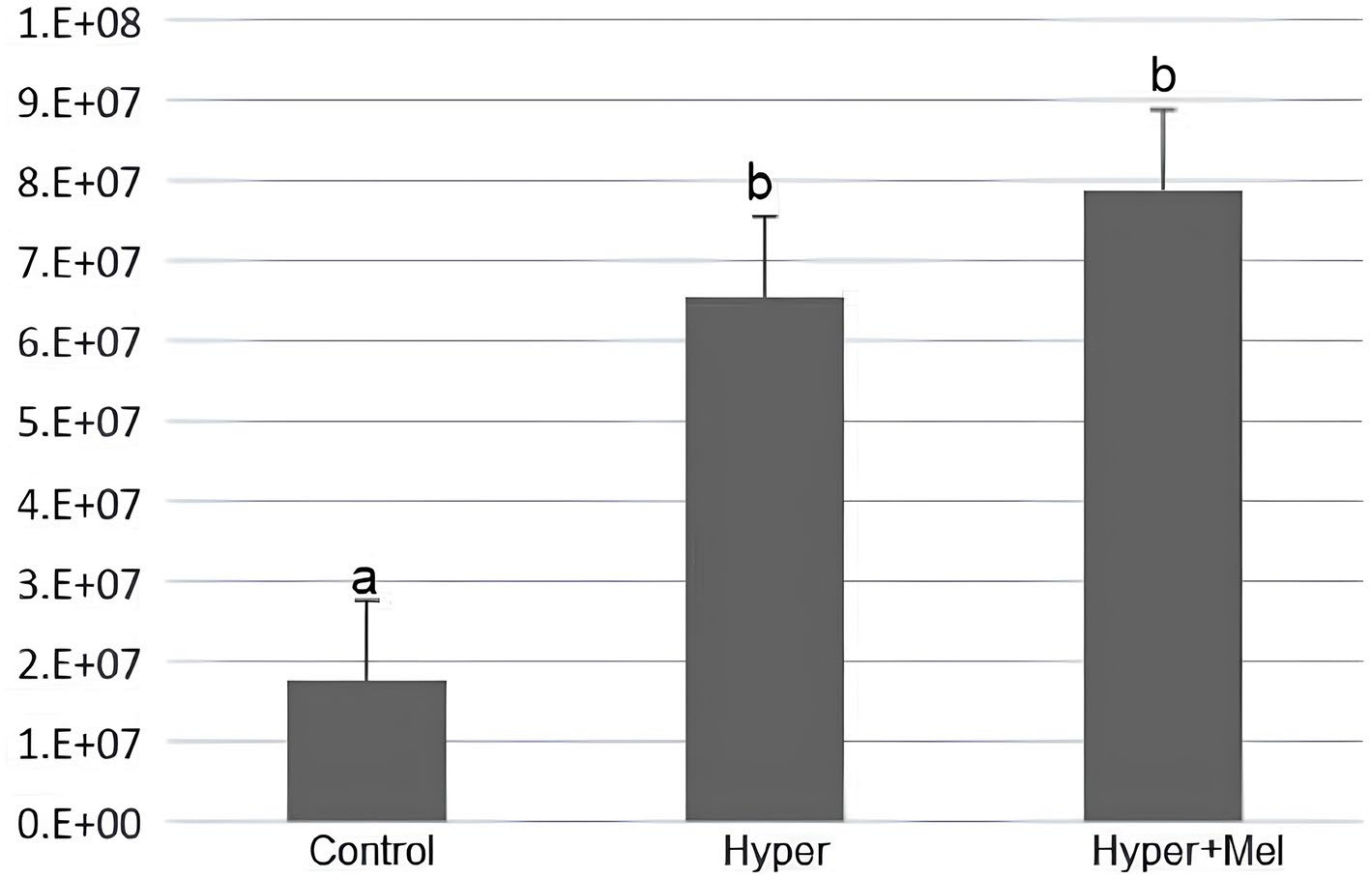
Analysis of PCNA in the liver of the experimental groups (*p* = 0.00). *Different letters indicate statistically significant differences (Kruskal–Wallis test followed by Dunn’s *post-hoc* test, *p* ≤ 0.05).

In the liver, PCNA analysis revealed a significant elevation in the Hyper group compared to the Control group. Notably, this increase persisted as statistically significant despite the presence of exogenous MEL (Figure 9).

### 3.7 Hormone assay

**Figure 10:**
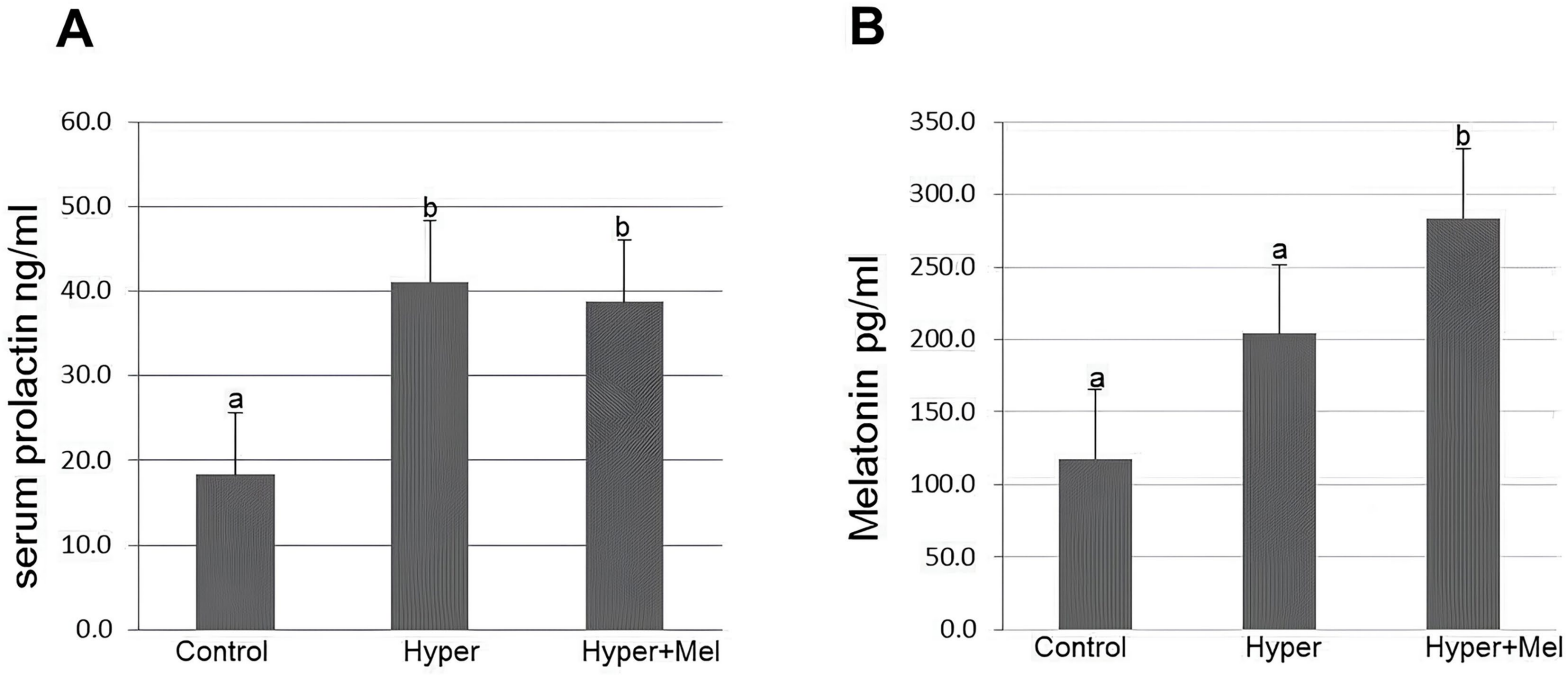
Serum PRL **(A)** and MEL **(B)** levels in the experimental groups (*n* = 8 per group). (a) Serum prolactin levels (*p* = 0.004). (b) Serum melatonin levels (*p* = 0.001). *Different letters indicate statistically significant differences (Kruskal–Wallis test followed by Dunn’s *post-hoc* test, *p* ≤ 0.05).

Serum PRL concentrations showed significantly higher values in the Hyper and Hyper+MEL groups than the Control group, whereas MEL concentrations exhibited a significant increase only in the Hyper+MEL group (Figure 10).

## 4 Discussion

Studies report that hyperprolactinemia is associated with increased food intake and, consequently, weight gain (23, 24), consistent with the findings of this study, as the Hyper group showed higher body weight than the Control group. However, the mechanism by which hyperprolactinemia may lead to weight gain is still poorly understood among researchers (25), with stimulation of lipogenesis being a possible explanation (26).

In lipogenesis, PRL influences by enhancing the mechanism of leptin synthesis and secretion (27), a hormone responsible for regulating appetite and metabolic homeostasis (28). Excess leptin (hyperleptinemia) in the body can lead to reduced sensitivity to this hormone (leptin resistance), resulting in hyperphagia, decreased energy expenditure, and weight gain (29).

Nevertheless, excessive weight gain can contribute to overweight and obesity, leading to other problems such as metabolic syndrome, obstructive sleep apnea, cardiovascular diseases, and type II diabetes (30). In the literature, the elevation of serum PRL levels in humans has been associated with weight gain in individuals with prolactinoma as well as those using antipsychotic medications (31, 32). Thus, overweight and obesity may be considered additional clinical manifestations of hyperprolactinemia (33).

On the other hand, the Hyper+MEL group showed body weight values close to those of the Control group, suggesting a possible influence of MEL on energy metabolism. However, this finding should be interpreted with caution, as it does not allow a definitive conclusion regarding a protective effect of MEL, since a previous study (34) reported that oral MEL administration (4 mg/kg) for 6 weeks did not reduce the total body weight of obese rats.

In contrast, some studies report that MEL may affect body mass. These discrepancies may be related to differences in experimental conditions, particularly dosage, duration of treatment, route of administration, and metabolic status. A previous study (35), investigating obese animals supplemented with MEL in drinking water (100 mg/kg) for 8 weeks, described a significant reduction of approximately 5% in total body weight, as well as reductions of 53 and 41% in visceral and subcutaneous adipose pads, respectively.

In a previous study (36), Sprague Dawley rats were fed a high-carbohydrate diet and supplemented with MEL via gavage (30 mg/kg) for 3 weeks, and the results also demonstrated a significant decrease of approximately 5% in total body weight.

Evidence indicates that MEL acts on the activation of brown adipose tissue (37, 38), increasing thermogenic activity through UCP-1 protein (39) and consequently increasing energy expenditure (40), which may contribute to the reduction in body weight in animals treated with MEL. The result of this study is consistent with the findings of previous studies (35, 36), as the Hyper+MEL experimental group showed a similar average to the Control group.

The histological examination of the stomach revealed dilation of the glandular lumen in the Hyper and Hyper+MEL groups compared to the Control group, and this dilation was also indicated by histomorphometric analysis. Nevertheless, the histological examination of the duodenum did not show any alterations. There are few studies in the literature reporting the possible effects of hyperprolactinemia on the tissues of these organs, highlighting an important gap that the present study sought to address.

Regarding the histological analysis of the liver, studies report that leptin resistance can negatively interfere with the lipolysis process, inhibiting fatty acid oxidation and consequently contributing to fat accumulation in tissues (41, 42), which may help explain the accumulation of lipid droplets (microsteatosis) along the liver tissue in the Hyper group of this study.

In the literature, leptin resistance has already been related to cases of non-alcoholic fatty liver disease (NAFLD). After a meta-analysis conducted with 33 studies in humans, it was concluded that patients with NAFLD have higher circulating levels of leptin than healthy individuals (43).

In this context, studies have reported that the hormone MEL can attenuate NAFLD. In the investigation conducted by a previous study (44), rats were fed a methionine/choline-deficient diet (MCDD) and treated with MEL intraperitoneally (50 mg/kg) for 4 weeks, and the results showed a reduction in NAFLD caused by MCDD.

Similarly, another previous study (45), evaluating the liver tissue of patients with NAFLD treated with MEL at a dose of 2.5 mg/day for 14 months, also reported a decrease in this disorder. The potential effect of MEL in reducing NAFLD may be related to its antioxidant activity since oxidative stress is characterized as one of the factors involved in the pathogenesis of this abnormality (46–49). However, an accumulation of lipid droplets (microsteatosis) was also observed throughout the liver tissue in the Hyper+MEL group of this study, suggesting that exogenous MEL was not able to attenuate liver damage in hyperprolactinemic rats under the conditions of this study.

Taken together, these findings suggest that hyperprolactinemia appears to promote systemic metabolic and morphological changes across multiple organs, while MEL may exhibit context-dependent effects. Although this hormone is widely recognized for its antioxidant (14) and anti-inflammatory (15) properties, its biological activity may vary according to factors such as dose, route of administration, metabolic status, and tissue-specific signaling. In endocrine disturbances such as hyperprolactinemia, changes in hormonal balance and cellular signaling pathways can modify tissue responsiveness to MEL, while its interaction with different molecular targets, including MT1/MT2 receptors, antioxidant systems, and homeostatic regulators, may lead to distinct outcomes, modulated by the local physiological environment. These mechanisms may help explain why exogenous MEL appeared to attenuate certain alterations in the present study but failed to prevent or reverse others.

In histochemical analysis, the means regarding the areas stained by PAS, a dye that highlights neutral mucin secretory vesicles in goblet cells, revealed that the Hyper group had a significantly lower value than the Control group. According to the literature, individuals with hyperprolactinemia experience abnormalities in carbohydrate metabolism (33). However, studies reporting the mechanisms of action of this condition on carbohydrate metabolism and how these contribute to metabolic dysfunctions are still scarce.

Mucins are the main components of the mucus that coats the intestinal surface. This mucus lubricates the epithelial mucosa and represents the first line of defense against the invasion of pathogenic microorganisms and external molecules, preventing the direct entry of agents considered harmful into epithelial cells (50, 51). Based on these results, hyperprolactinemia may be associated with alterations in mucin metabolism, thereby affecting mucus production and compromising intestinal protection. Nevertheless, this interpretation should be regarded as preliminary, since the underlying biological mechanisms remain unclear and further studies are required to confirm this hypothesis.

On the other hand, the Hyper+MEL group showed a significantly higher average, demonstrating a value close to that of the Control group. The literature has already reported the positive influence of the MEL hormone on carbohydrate metabolism (52). In a previous study (53), weaning mice were supplemented with MEL in drinking water (0.2 mg/mL) for 2 weeks, and the results demonstrated that in the last portion of the small intestine, i.e., in the ileum, supplementation of this hormone affected mucin2 mRNA expression. The data from this study are consistent with previous findings, since the Hyper+MEL experimental group showed a similar average to the Control group.

Histomorphometric analysis of the duodenum revealed a significant increase in the height of intestinal villi and microvilli in the Hyper and Hyper+MEL groups compared to the Control group. Villi, along with microvilli, are responsible for increasing the surface area of intestinal absorption (54). The small intestine, like other organs, undergoes structural and functional changes during development. However, as age advances, these changes can be increased or decreased, depending on environmental influences (55). According to another study (56), the amount of food ingested affects epithelial growth, both directly and by stimulating the secretion of enterohormones. Thus, the intestinal mucosa is hypertrophied when there is food overload and atrophied in the absence of food (57).

In light of this, the increase in villus and microvillus heights observed in the Hyper and Hyper+MEL groups may be associated with increased food intake, which may be driven by hyperprolactinemia. Nevertheless, this explanation should be considered exploratory, as previous studies have reported divergent findings, such as that reported by Muller and Dowling (8), which observed that hyperprolactinemia did not cause alterations in intestinal villus height in the jejunum and ileum of lactating rats.

On the other hand, the histomorphometric analysis of the liver revealed a significant decrease in the count of KCs in the Hyper group than the Control group. Even in the presence of exogenous MEL, this reduction was statistically significant. KCs, known as resident liver macrophages, are located within the hepatic sinusoids and contribute to maintaining homeostasis and regulating the immune response in this organ. In other words, KCs have intense phagocytic activity with the ability to, for example, metabolize senescent red blood cells, digest hemoglobin, and destroy pathogens that for some reason enter the portal blood (58). Thus, our data suggest that hyperprolactinemia may result in liver alterations through the reduction of KCs. However, the role of MEL remains unclear in this context, as no protective effect was observed in our model. This finding contradicts Muller and Dowling (59), who also analyzed the livers of rats induced with hyperprolactinemia and treated with MEL subcutaneously (200 μg/100 g) for 30 days, and reported that the condition did not alter the number of KCs, since the values for this parameter did not show a statistically significant difference between the experimental groups investigated. Therefore, treatment duration may be an important factor contributing to these differences in the possible changes caused by hyperprolactinemia.

Beyond the counting of KCs, the histomorphometric analysis of the liver also included the quantification of binucleated hepatocytes. Regarding this parameter, the Hyper+MEL group showed a significantly higher value than the Control group, reflecting a tendency toward cell proliferation. Although the Hyper group did not differ significantly from the Control group, it also exhibited higher values, indicating a proliferative propensity.

In terms of nonlinear analysis, the FD of hepatic sinusoids was significantly higher in the Hyper group than in the Control group. This increase remained statistically significant even in the presence of exogenous MEL. Irregular structures analyzed by the FD method are quantified based on their complexity, with higher FD values indicating more complex images (60).

These findings suggest a possible alteration in the anatomy of hepatic sinusoids due to hyperprolactinemia, increasing their complexity and irregularity. However, given the lack of prior validation of FD and lacunarity analyses in this tissue, these results should be interpreted as exploratory and preliminary. In both the Hyper and Hyper+MEL groups, hepatic sinusoids appeared to be distributed in a more disproportionate manner compared to the Control group, highlighting the increased complexity/irregularity indicated by the FD result. Nevertheless, this potential anatomical alteration of hepatic sinusoids linked to hyperprolactinemia was not indicated by lacunarity, a complementary fractal parameter that describes the heterogeneity and spatial distribution of gaps within a structure (60), as the values of this parameter did not show a statistically significant difference between the groups investigated in this study.

On the other hand, results related to FD and lacunarity of duodenal goblet cells did not show a statistically significant difference between the groups studied. In the literature, there are no previous reports of using FD and lacunarity to detect changes in hepatic sinusoids and duodenal goblet cells in hyperprolactinemic rats treated with exogenous MEL, representing an approach that has not been previously reported in this experimental context. Future studies are required to validate the application of FD and lacunarity analyses in these tissues and to confirm the biological relevance of the observed patterns.

Immunohistochemical analysis revealed that PCNA expression varied according to the tissue examined. In the stomach, only the Hyper+MEL group showed a significant increase than in the Control group, while in the liver, both Hyper and Hyper+MEL groups exhibited higher PCNA immunolabeling. In contrast, in the duodenum, both hyperprolactinemic groups demonstrated reduced PCNA expression relative to the Control group, differing from previous reports of hyperplasia in the jejunum and ileum (8). These findings support the notion that PRL can stimulate cellular proliferation, but also suggest that its effects are tissue-dependent. The association between hyperprolactinemia and increased risk of oncological pathologies (10) is partially consistent with our results, particularly in the liver and stomach. However, the absence of changes in the Hyper group in the stomach, together with the reduction observed in the duodenum, indicates that PRL’s influence on cell proliferation in the digestive system is complex and warrants further investigation.

Evidence suggests that MEL has an antiproliferative effect (61). Exogenous MEL in this study was not sufficient to reverse the pronounced cell proliferation demonstrated in the stomach and liver of hyperprolactinemic rats.

However, despite the antiproliferative effect of MEL not being revealed in this investigation, it has been demonstrated in other studies. A previous study (62) reported that the administration of MEL (200 mg/kg) in mice with oral cancer was able to decrease the cell proliferation rate and, consequently, tumor volume, as well as reduce the expression of genes associated with tumor development.

Another study (63) showed that MEL significantly inhibited the cell proliferation index in lung cancer, revealing the anticancer potential of this hormone.

Regarding PRL hormonal dosage, animals in the Hyper group showed higher concentrations than those in the Control group, confirming that DOMP is associated with increased serum PRL concentrations, leading to hyperprolactinemia, as previously reported by several other studies (19, 64, 65). Furthermore, it is known that the elevation of PRL hormone levels stimulated by DOMP lasts at least 72 h, without developing tolerance by the tuberoinfundibular dopaminergic system, which is responsible for controlling the synthesis and secretion of PRL (66). Thus, the morphological changes observed in the investigated organs are likely associated with hyperprolactinemia, although further research is needed to clarify the underlying mechanisms.

Although evidence suggests that MEL may modulate PRL release, our findings did not demonstrate such an effect, as PRL levels remained elevated in the Hyper+MEL group. This modulatory role has been proposed because MEL receptors have been identified in the pars tuberalis of the pituitary gland, a region involved in the photoperiodic regulation of PRL secretion (67, 68). Consistent with this mechanism, Martinet et al. (69) reported that administration of 100 μg of MEL into the bloodstream of pregnant female *Mustela vison* reduced circulating PRL levels.

On the other hand, a previous study (19) described that subcutaneous administration of MEL (200 μg/100 g) for 30 days was not able to reduce serum PRL concentrations in hyperprolactinemic rats. This finding is consistent with the results of this study, as the Hyper+MEL experimental group also showed higher serum PRL levels than the Control group. Due to the discrepant data, it becomes important to conduct more in-depth investigations regarding this parameter to establish more accurately and clearly the potential association between PRL and MEL.

Regarding the hormonal dosage of MEL, animals in the Hyper+MEL group showed higher serum levels than the Control and Hyper groups, confirming the circulation of exogenous MEL in their bodies. In the scientific field, MEL has been investigated as a possible therapy for various diseases due to its effects: neuroprotective, antioxidant, anti-inflammatory, anti-aging, anticancer, and gastroprotective (70). Additionally, another study (71) confirms that MEL is a substance with low toxicity, and no adverse effects have been observed from its use in adults, even with doses exceeding 300 mg. Multiple other studies confirm the safety and low toxicity of MEL (72, 73), but self-medication is not recommended, and administration without proper medical guidance should be avoided (74). Despite this, the exogenous MEL administered in this study did not demonstrate consistent protective effects across all evaluated parameters, perhaps due to the dosage or the treatment period, considering that the studies cited throughout this investigation adopted different therapeutic dosages and intervals. Standardizing dose and treatment duration across studies may help achieve more consistent and reproducible results and clarify the potential protective effects of MEL in the body. Although some statistically significant differences were observed across the evaluated parameters, the magnitude of these variations was relatively small and should therefore be interpreted with caution regarding their biological relevance in the context of the present findings.

### 4.1 Study limitations

Some limitations of this investigation should be acknowledged. The sample size was relatively small, albeit comparable to those used in similar studies involving endocrine and histological assessments in rodent models; thus, larger cohorts would provide greater statistical power and enable more robust detection of subtle differences. The experimental design did not include a group treated exclusively with MEL; therefore, even though the present findings allow evaluation of its administration in the context of hyperprolactinemia, its isolated physiological effects cannot be fully distinguished. The inclusion of a MEL-only group in subsequent research may help clarify these mechanisms. A potential influence of circadian rhythms on PRL and MEL secretion should also be considered. Despite blood collection being performed within a standardized time window, circadian variability cannot be completely excluded and may have influenced hormonal measurements. Correlation analyses between hormonal levels and tissue parameters were not performed, since the primary objective of this study was to compare experimental groups rather than to investigate hormonal-cellular associations; nevertheless, these relationships warrant further investigation. Fractal analysis was applied as a quantitative tool to assess tissue organization and structural complexity. While increasingly used in biological research, its interpretation in gastrointestinal and hepatic histology remains exploratory and requires additional validation. Finally, as the study was conducted exclusively in male rats, the findings cannot be directly extrapolated to women; accordingly, studies including both sexes are encouraged to enhance the generalizability of the results.

## 5 Conclusion

The present study suggests that DOMP-induced hyperprolactinemia is linked to morphological changes in the stomach, duodenum, and liver, as well as increased body weight in rats. Exogenous MEL treatment appeared to modulate body weight and histochemical parameters; however, in some analyses, signs of an intensification of these changes were observed. These findings indicate that the action of MEL may vary depending on the experimental context, exhibiting potentially beneficial effects under certain conditions while also being associated with adverse tissue responses in others. Further studies are needed to clarify the mechanisms underlying the interaction between exogenous MEL administration and DOMP-induced hyperprolactinemia and to better define the role of MEL in gastrohepatic alterations associated with this condition.

## Data Availability

The original contributions presented in the study are included in the article/supplementary material, further inquiries can be directed to the corresponding author.

## References

[1] Bugge K Papaleo E Haxholm GW Hopper JTS Robinson CV Olsen JG . A combined computational and structural model of the full-length human prolactin receptor. Nat Commun. (2016) 7:11578. doi: 10.1038/ncomms11578, 27174498 PMC4869255

[2] Vilar L Vilar CF Lyra R Freitas MC. Pitfalls in the diagnostic evaluation of hyperprolactinemia. Neuroendocrinology. (2019) 109:7–19. doi: 10.1159/000499694, 30889571

[3] Vilar L Abucham J Albuquerque JL Araujo LA Azevedo MF Boguszewski CL . Controversial issues in the management of hyperprolactinemia and prolactinomas – an overview by the neuroendocrinology department of the Brazilian Society of endocrinology and metabolism. Arch Endocrinol Metab. (2018) 62:236–63. doi: 10.20945/2359-3997000000032, 29768629 PMC10118988

[4] Molitch ME. Disorders of prolactin secretion. Endocrinol Metab Clin N Am. (2001) 30:585–610. doi: 10.1016/S0889-8529(05)70203-6 11571932

[5] Mah PM Webster J. Hyperprolactinemia: etiology, diagnosis, and management. Semin Reprod Med. (2002) 20:365–74. doi: 10.1055/s-2002-36709 12536359

[6] Mancini T Casanueva FF Giustina A. Hyperprolactinemia and prolactinomas. Endocrinol Metab Clin N Am. (2008) 37:67–99. doi: 10.1016/j.ecl.2007.10.013 18226731

[7] Vergani G Mayerhofer A Bartke A. Acute effects of rat growth hormone (GH), human GH and prolactin on proliferating rat liver cells in vitro: a study of mitotic behaviour and ultrastructural alterations. Tissue Cell. (1994) 26:457–65. doi: 10.1016/0040-8166(94)90029-9, 8073420

[8] Muller E Dowling RH. Prolactin and the small intestine. Effect of hyperprolactinemia on mucosal structure in the rat. Gut. (1981) 22:558–65. doi: 10.1136/gut.22.7.558, 7262630 PMC1419333

[9] Seretis C Seretis F Liakos N Pappas A Keramidakis D Gourgiotis S . Constipation-predominant irritable bowel syndrome associated with hyperprolactinemia. Case Rep Gastroenterol. (2011) 5:523–7. doi: 10.1159/000331806, 22087083 PMC3214685

[10] Berinder K Akre O Granath F Hulting AL. Cancer risk in hyperprolactinemia patients: a population-based cohort study. Eur J Endocrinol. (2011) 165:209–15. doi: 10.1530/EJE-11-0076, 21602317

[11] Lanoix D Beghdadi H Lafond J Vaillancourt C. Human placental trophoblasts synthesize melatonin and express its receptors. J Pineal Res. (2008) 45:50–60. doi: 10.1111/j.1600-079X.2008.00555.x, 18312298

[12] Bubenik GA. Gastrointestinal melatonin: localization, function, and clinical relevance. Dig Dis Sci. (2002) 47:2336–48. doi: 10.1023/a:1020107915919, 12395907

[13] Konturek SJ Konturek PC Brzozowski T Bubenik GA. Role of melatonin in upper gastrointestinal tract. J Physiol Pharmacol. (2007) 58:23–52. 18212399

[14] Li R Luo X Li L Peng Q Yang Y Zhao L . The protective effects of melatonin against oxidative stress and inflammation induced by acute cadmium exposure in mice testis. Biol Trace Elem Res. (2016) 170:152–64. doi: 10.1007/s12011-015-0449-6, 26224376

[15] Carloni S Favrais G Saliba E Albertini MC Chalon S Longini M . Melatonin modulates neonatal brain inflammation through endoplasmic reticulum stress, autophagy, and miR-34a/silent information regulator 1 pathway. J Pineal Res. (2016) 61:370–80. doi: 10.1111/jpi.12354, 27441728

[16] Tamura H Kawamoto M Sato S Tamura I Maekawa R Taketani T . Long-term melatonin treatment delays ovarian aging. J Pineal Res. (2017) 62:e12381. doi: 10.1111/jpi.12381, 27889913

[17] Chen Z Lei L Wen D Yang L. Melatonin attenuates palmitic acid-induced mouse granulosa cell apoptosis via endoplasmic reticulum stress. J Ovarian Res. (2019) 12:1–12. doi: 10.1186/s13048-019-0519-z, 31077207 PMC6511168

[18] Chen CQ Fichna J Bashashati M Li YY Storr M. Distribution, function, and physiological role of melatonin in the lower gut. World J Gastroenterol. (2011) 17:3888–98. doi: 10.3748/wjg.v17.i34.3888, 22025877 PMC3198018

[19] Silva EFA Gomes JAS Oliveira MLF Noya AGAFC Magalhães CP Silva JV . Protective effect of exogenous melatonin on testicular histopathology and histomorphometry of adult rats with domperidone-induced hyperprolactinemia. Reprod Biol. (2023) 23:100791. doi: 10.1016/j.repbio.2023.100791, 37517145

[20] Conselho Nacional de Controle de Experimentação Animal (CONCEA). Diretriz brasileira para o cuidado e a utilização de animais para fins científicos e didáticos (DBCA). Brasília: Ministério da Ciência, Tecnologia e Inovação (2013).

[21] American Veterinary Medical Association (AVMA). AVMA Guidelines for the Euthanasia of Animals. 2020th ed. Schaumburg: AVMA (2020).

[22] Richter HG Hansell JA Raut S Giussani DA. Melatonin improves placental efficiency and birth weight and increases the placental expression of antioxidant enzymes in undernourished pregnancy. J Pineal Res. (2009) 46:357–64. doi: 10.1111/j.1600-079X.2009.00671.x, 19552758

[23] Moore BJ Gerardo-Gettens T Horwitz BA Stern JS. Hyperprolactinemia stimulates food intake in the female rat. Brain Res Bull. (1986) 17:563–9. doi: 10.1016/0361-9230(86)90226-1, 3779456

[24] Byatt JC Staten NR Salsgiver WJ Kostelc JG Collier RJ. Stimulation of food intake and weight gain in mature female rats by bovine prolactin and bovine growth hormone. Am J Phys. (1993) 264:E986–92. doi: 10.1152/ajpendo.1993.264.6.E986, 8333524

[25] Shibli-Rahhal A Schlecte J. The effects of hyperprolactinemia on bone and fat. Pituitary. (2009) 12:96–104. doi: 10.1007/s11102-008-0097-3, 18338266

[26] Doknic M Pekic S Zarkovic M Medic-Stojanoska M Dieguez C Casanueva F . Dopaminergic tone and obesity: an insight from prolactinomas treated with bromocriptine. Eur J Endocrinol. (2002) 147:77–84. doi: 10.1530/eje.0.1470077, 12088923

[27] Considine RV Sinha MK Heiman ML Kriauciunas A Stephens TW Nyce MR . Serum immunoreactive-leptin concentrations in normal-weight and obese humans. N Engl J Med. (1996) 334:292–5. doi: 10.1056/NEJM199602013340503, 8532024

[28] Gualillo O Lago F Garcia M Menendez C Senaris R Casanueva FF . Prolactin stimulates leptin secretion by rat white adipose tissue. Endocrinology. (1999) 140:5149–53. doi: 10.1210/endo.140.11.7147, 10537143

[29] Reseland JE Anderssen SA Solvoll K Hjermann I Urdal P Holme I . Effect of long-term changes in diet and exercise on plasma leptin concentrations. Am J Clin Nutr. (2001) 73:240–5. doi: 10.1093/ajcn/73.2.240, 11157319

[30] Araújo GB Figueiredo IHS Araújo BS Oliveira IMM Dornelles C Aguiar JRV . Relação entre sobrepeso e obesidade e o desenvolvimento ou agravo de doenças crônicas não transmissíveis em adultos. Res Soc Dev. (2022) 11:e50311225917. doi: 10.33448/rsd-v11i2.25917

[31] Baptista T Lacruz A Meza T Contreras Q Delgado C Mejias MA . Antipsychotic drugs and obesity: is prolactin involved? Can J Psychiatr. (2001) 46:829–34. doi: 10.1177/070674370104600906, 11761634

[32] Schmid C Goede DL Hauser RS Brandle M. Increased prevalence of high body mass index in patients presenting with pituitary tumours: severe obesity in patients with macroprolactinoma. Swiss Med Wkly. (2006) 136:254–8. doi: 10.4414/smw.2006.10955, 16708311

[33] Ben-Jonathan N Hugo ER Brandebourg TD Lapensee CR. Focus on prolactin as a metabolic hormone. Trends Endocrinol Metab. (2006) 17:110–6. doi: 10.1016/j.tem.2006.02.005 16517173

[34] Nduhirabandi F Huisamen B Strijdom H Blackhurst D Lochner A. Short-term melatonin consumption protects the heart of obese rats independent of body weight change and visceral adiposity. J Pineal Res. (2014) 57:317–32. doi: 10.1111/jpi.12171, 25187154

[35] Favero G Stacchiotti A Castrezzati S Bonomini F Albanese M Rezzani R . Melatonin reduces obesity and restores adipokine patterns and metabolism in obese (Ob/Ob) mice. Nutr Res. (2015) 35:891–900. doi: 10.1016/j.nutres.2015.07.001, 26250620

[36] Prunet-Marcassus B Desbazeille M Bros A Louche K Delagrange P Renard P . Melatonin reduces body weight gain in Sprague Dawley rats with diet-induced obesity. Endocrinology. (2003) 144:5347–52. doi: 10.1210/en.2003-0693, 12970162

[37] Cannon B Nedergaard J. Brown adipose tissue: function and physiological significance. Physiol Rev. (2004) 84:277–359. doi: 10.1152/physrev.00015.2003 14715917

[38] Tan DX Manchester LC Fuentes-Broto L Paredes SD Reiter RJ. Significance and application of melatonin in the regulation of brown adipose tissue metabolism: relation to human obesity. Obes Rev. (2011) 12:167–88. doi: 10.1111/j.1467-789X.2010.00756.x, 20557470

[39] Halpern B Mancini MC Bueno C Barcelos IP Melo ME Lima MS . Melatonin increases brown adipose tissue volume and activity in patients with melatonin deficiency: a proof-of-concept study. Diabetes. (2019) 68:947–52. doi: 10.2337/db18-0956, 30765337

[40] Nottar CL Trentin STF Grassiolli S Casaril KBPB. Tecido adiposo marrom: um aliado na luta contra obesidade. Acta Elit Salutis. (2021) 5:1–12. doi: 10.48075/aes.v5i1.27718

[41] Romero CEM Zanesco A. The role of leptin and ghrelin on the genesis of obesity. Rev Nutr. (2006) 19:85–91. doi: 10.1590/S1415-52732006000100009

[42] Boutari C Perakakis N Mantzoros CS. Association of adipokines with development and progression of nonalcoholic fatty liver disease. Endocrinol Metab. (2018) 33:33–43. doi: 10.3803/EnM.2018.33.1.33, 29589386 PMC5874193

[43] Polyzos SA Aronis KN Kountouras J Raptis DD Vasiloglou MF Mantzoros CS. Circulating leptin in non-alcoholic fatty liver disease: a systematic review and meta-analysis. Diabetology. (2016) 59:30–43. doi: 10.1007/s00125-015-3769-3, 26407715

[44] Tahan V Atug O Akin H Eren F Tahan G Tarcin O . Melatonin ameliorates methionine – and choline-deficient diet – induced nonalcoholic steatohepatitis in rats. J Pineal Res. (2009) 46:401–7. doi: 10.1111/j.1600-079X.2009.00676.x, 19552763

[45] Celinski K Konturek PC Slomka M Cichoz-Lach H Brzozowski T Konturek SJ . Effects of treatment with melatonin and tryptophan on liver enzymes, parameters of fat metabolism, and plasma levels of cytokines in patients with non-alcoholic fatty liver disease: 14- month follow-up. J Physiol Pharmacol. (2014) 65:75–82. 24622832

[46] Zhao F Liu ZQ Wu D. Antioxidative effect of melatonin on DNA and erythrocytes against free-radical-induced oxidation. Chem Phys Lipids. (2008) 151:77–84. doi: 10.1016/j.chemphyslip.2007.10.002, 17996197

[47] Larter CZ Chitturi S Heydet D Farrell GC. A fresh look at the pathogenesis of NASH. Part 1: the metabolic movers. J Gastroenterol Hepatol. (2010) 25:672–90. doi: 10.1111/j.1440-1746.2010.06253.x, 20492324

[48] Ono M Okamoto N Saibara T. The latest idea in NAFLD/NASH pathogenesis. Clin J Gastroenterol. (2010) 3:263–70. doi: 10.1007/s12328-010-0182-9, 26190482

[49] McCarty MF. Full-spectrum antioxidant therapy featuring astaxanthin coupled with lipoprivic strategies and salsalate for management of nonalcoholic fatty liver disease. Med Hypotheses. (2011) 77:550–6. doi: 10.1016/j.mehy.2011.06.029, 21764223

[50] Camilleri M Madsen K Spiller R Meerveld BGV Verne GN. Intestinal barrier function in health and gastrointestinal disease. Neurogastroenterol Motil. (2012) 24:503–12. doi: 10.1111/j.1365-2982.2012.01921.x, 22583600 PMC5595063

[51] Vancamelbeke M Vermeire S. The intestinal barrier: a fundamental role in health and disease. Expert Rev Gastroenterol Hepatol. (2017) 11:821–34. doi: 10.1080/17474124.2017.1343143, 28650209 PMC6104804

[52] Seraphim PM Sumida DH Nishide FY Lima FB Neto JC Machado UFA. Pineal gland and carbohydrate metabolism. Arq Bras Endocrinol Metab. (2000) 44:331–8. doi: 10.1590/S0004-27302000000400009

[53] Ren W Wang P Yan J Liu G Zeng B Hussain T . Melatonin alleviates weanling stress in mice: involvement of intestinal microbiota. J Pineal Res. (2018) 64:e12448. doi: 10.1111/jpi.12448, 28875556

[54] Desesso JM Jacobson CF. Anatomical and physiological parameters affecting gastrointestinal absorption in humans and rats. Food Chem Toxicol. (2001) 39:209–28. doi: 10.1016/s0278-6915(00)00136-8, 11278053

[55] Pacha J. Development of intestinal transport function in mammals. Physiol Rev. (2000) 80:1633–67. doi: 10.1152/physrev.2000.80.4.1633 11015621

[56] Johnson LR. Regulation of gastrointestinal mucosal growth. Physiol Rev. (1988) 68:456–502. doi: 10.1152/physrev.1988.68.2.456 3282244

[57] Pluske JR Hampson DJ Williams IH. Factors influencing the structure and function of the small intestine in the weaned pig: a review. Livest Prod Sci. (1997) 51:215–36. doi: 10.1016/S0301-6226(97)00057-2

[58] Kubes P Jenne C. Immune responses in the liver. Annu Rev Immunol. (2018) 36:247–77. doi: 10.1146/annurev-immunol-051116-052415 29328785

[59] Guedes GHF Costa MAS Carvalho JM Oliveira MLF Tenorio FCAM. Efeito da Melatonina na Morfometria e Histoquímica do Fígado de Ratos Induzidos à Hiperprolactinemia Anais IV CONAPESC. Campina Grande: Realize Editora (2019).

[60] Moreira RD Moriel AR Murta-Junior LO Neves LA Godoy MF. Dimensão fractal na quantificação do grau de rejeição celular miocárdica pós-transplante cardíaco. Rev Bras Cir Cardiovasc. (2011) 26:155–63. doi: 10.1590/S0102-76382011000200004, 21894404

[61] Souza EM Feitosa PWG Rodrigues MRB Teles RB Silva MMB Ribeiro CAC. A Fisiofarmacologia da Melatonina no Desenvolvimento e Terapêutica do Câncer. Id on Line Rev Psic. (2022) 16:754–77. doi: 10.14295/idonline.v16i60.3459

[62] Hsieh MJ Lin CW Su SC Reiter RJ Chen AWG Chen MK . Effects of miR-34b/miR-892a upregulation and inhibition of ABCB1/ABCB4 on melatonin-induced apoptosis in VCR-resistant oral cancer cells. Mol Ther Nucleic Acids. (2020) 19:877–89. doi: 10.1016/j.omtn.2019.12.022, 31982774 PMC6994412

[63] Ma Z Liu D Di S Zhang Z Li W Zhang J . Histone deacetylase 9 downregulation decreases tumor growth and promotes apoptosis in non- small cell lung cancer after melatonin treatment. J Pineal Res. (2019) 67:e12587. doi: 10.1111/jpi.12587, 31090223

[64] Gomez-Ochoa P Castillo JA Gascon M Zarate JJ Alvarez F Couto CG. Use of domperidone in the treatment of canine visceral leishmaniasis: a clinical trial. Vet J. (2009) 179:259–63. doi: 10.1016/j.tvjl.2007.09.014, 18023375

[65] Ochoa-Amaya JE Malucelli BE Cruz-Casallas PE Nasello AG Felicio LF Carvalho-Freitas MIR. Acute and chronic stress and the inflammatory response in hyperprolactinemic rats. Neuroimmunomodulation. (2010) 17:386–95. doi: 10.1159/000292063, 20516720

[66] Donato J Jr Frazão R. Interactions between prolactin and kisspeptin to control reproduction. Arch Endocrinol Metab. (2016) 60:587–95. doi: 10.1590/2359-3997000000230, 27901187 PMC10522168

[67] Soares-Junior JM Masana MI Ersahin C Dubocovich ML. Functional melatonin receptors in rat ovaries at various stages of the estrous cycle. Pharmacol Exp Ther. (2003) 306:694–702. doi: 10.1124/jpet.103.049916, 12721330

[68] Lopez BD Rodriguez ED Urquijo C Alvarez CF. Melatonin influences on the neuroendocrine-reproductive axis. Ann N Y Acad Sci. (2005) 1057:337–64. doi: 10.1196/annals.1356.026, 16399905

[69] Martinet L Allain D Meunier M. Regulation in pregnant mink (*Mustela vison*) of plasma progesterone and prolactin concentrations and regulation of the onset of the spring moult by daylight ratio and melatonin injections. Can J Zoo. (1983) 61:1959–63. doi: 10.1139/z83-257

[70] Silva AR Silva DM Gomes IC Gomes ATA. Self-medication and the use of melatonin: integrative literature review. Braz J Health Rev. (2021) 4:21460–83. doi: 10.34119/bjhrv4n5-235

[71] Glanzmann R Moreira LF Marques AS Silva KC Soares VCGO. Uso da melatonina como indutor do sono: uma revisão bibliográfica. Rev Uningá. (2019) 56:157–67. doi: 10.46311/2318-0579.56.eUJ2094

[72] Costa RM Martins IS. Melatonina na insônia primária: quais as evidências? Rev Bras Med Fam Comunidade. (2016) 11:1–9. doi: 10.5712/rbmfc11(38)845

[73] Silva-Junior PR Cabral HR Gomes ALOR Teofilo PBE Oliveira TKB. Melatonina exógena e seus efeitos metabólicos: revisão da literatura. Anais Fac Med Olinda. (2019) 1:45–8. doi: 10.56102/afmo.2019.60

[74] Vainer AM Rocha VS Juvenale M. Melatonina e sistema imune: uma relação com duas vias regulatórias. Brazilian J Health Rev. (2021) 4:2906–29. doi: 10.34119/bjhrv4n1-234

